# Being alone and expectations lost: a critical realist study of maternal depression in South Western Sydney

**DOI:** 10.1186/s40064-015-1492-7

**Published:** 2015-11-14

**Authors:** John G. Eastwood, Lynn A. Kemp, Bin B. Jalaludin

**Affiliations:** Community Paediatrics, Sydney Local Health District, Croydon Community Health Centre, 24 Liverpool Road, Croydon, NSW 2132 Australia; School of Public Health and Community Medicine, The University of New South Wales, Sydney, NSW 2052 Australia; School of Women’s and Children’s Health, The University of New South Wales, Sydney, NSW 2052 Australia; Ingham Institute of Applied Medicine, The University of New South Wales, Liverpool, NSW 2170 Australia; School of Public Health, The University of Sydney, Sydney, NSW 2006 Australia; School of Medicine, Griffith University, Gold Coast, QLD 4222 Australia; School of Nursing and Midwifery, Western Sydney University, Campbelltown, NSW 2560 Australia

**Keywords:** Postnatal depression, Critical realism, Mixed methods, Isolation, Expectations

## Abstract

The study reported here is part of a critical realist multilevel study. It seeks to identify and explain complex perinatal contextual social and psychosocial mechanisms that may influence the developmental origins of health and disease, with a focus on the role of postnatal depression. The aims of the greater study are to: (1) describe the phenomenon of postnatal depression in South Western Sydney; and (2) identify mechanisms that would add to our understanding of the psycho-social causes of maternal depression. This paper will move beyond our previous quantitative descriptions of individual-level predictors of depressive symptoms by seeking the views of local mothers and practitioners, to explain the mechanisms that might be involved. The study was set in South Western Sydney, New South Wales, Australia. An Explanatory Theory Building Method was used. The previously reported quantitative study was a non-linear principal component analysis and logistic regression study of 15,389 months delivering in 2002 and 2003. This intensive qualitative study used open coding of interviews, of seven practitioners and three naturally occurring mothers groups, to enable maximum emergence. The theoretical concepts identified were: attachment and nurturing, infant temperament, unplanned pregnancy and sole parenthood, support for mothers, access to services, stress, financial hardship, isolation and marginalisation, mothers’ “loss of control” and “power”, and expectations and dreams. *Being alone* and *expectations lost emerged* as possible triggers of stress and depression for mothers. These findings might also apply to others who have their dreams shattered during life’s transitions. In these situations social and cultural context can either nurture and support or marginalise and isolate. The challenge for policy and practice is to support mothers and their partners during the transition to parenthood within a challenging social and material context.

## Background

The study reported here is part of a critical realist mixed method multilevel study that seeks to identify and explain complex perinatal contextual social and psychosocial mechanisms that may influence the developmental origins of health and disease with a focus on the role of postnatal depression (Eastwood et al. 2014). This paper aims to move beyond our previous quantitative descriptions of individual-level predictors of depressive symptoms by seeking the views of local mothers and practitioners to explain the mechanisms which might be involved.

Current evidence, from both human and animal studies suggest that perinatal adversity, stress, and inflammation; genetic and epigenetic changes; and hormonal dysregulation, including changes in hypothalamic–pituitary–adrenal axis, play key roles in the development of perinatal reproductive mood disorders (Gluckman and Hanson 2006; Matthews and Meaney 2005; Meaney 2010; Osborne and Monk 2013; Monk et al. 2012; Meltzer-Brody 2011). There has been less emphasis given, however, to the role played by maternal depression in developmental origins of health and disease (DOHD) and intergenerational cycles of psychopathology (ICP). Postnatal maternal depression has been consistently demonstrated to have an adverse impact on maternal-infant attachment and interaction (Beck 1995; Murray et al. 1996; Martins and Gaffan 2000) with resulting child language, cognitive, behavioural and psychological problems (Cogill et al. 1986; Downey and Coyne 1990; Gelford and Teti 1990; Murray et al. 1996; Cummings and Davies 1994; Sohr-Preston and Scaramella 2006).

The prevalence of postnatal maternal depression in high income countries is between 13 and 20 percent (Gavin et al. 2005) with a recent Australian study reporting a prevalence of 15.5 percent (Buist et al. 2008). Using the Edinburgh Postnatal Depression Scale (EPDS) in a large Sydney study, we reported EPDS >9 of 12.2 percent and EPDS >12 of 6.8 percent (Eastwood et al. 2011).

We have recently reported on the multilevel spatial distribution of depressive symptoms among migrant mothers in South Western Sydney (Eastwood et al. 2013). That study suggested that migrant mothers who were socially isolated were at greater risk of experiencing depressive symptoms. Previous Australian studies have also found high rates of maternal depression among migrant women (Williams and Carmichael 1985; Brown and Lumley 2000; Brown et al. 1994; Lansakara et al. 2010). High rates of postnatal depression among migrants have also been found among Pacific Island mothers in Auckland, New Zealand (Abbott and Williams 2006), Canadian immigrants, asylum seekers and refugees (Stewart et al. 2008; Sword et al. 2006; Dennis et al. 2009), London ethnic minorities (Onozawa et al. 2003) and Latinas or Hispanic US mothers (Diaz et al. 2007; Beck et al. 2005). Almond (2009) has recently drawn attention to the global public health implications of postnatal depression with the greatest burden being experienced by low- to middle-income countries.

As we have previously observed “the significance of these findings is complicated by the wide international cross-cultural variation in the definition and understanding of postnatal depression and depressive symptoms” (Eastwood et al. 2013). Halbreich and Karkum (2006) undertook a review of 143 studies from 40 countries and found a wide range in reported rates. Bina (2008) analysed 70 studies on culture and postnatal depression. Social support was identified as important but specifically the review highlighted the importance of the woman’s perception of support (Bina 2008).

It has been proposed that depression is caused by psychological stress (Kinderman 2005; Stone et al. 2008) but this tendency of stress to cause depression is almost certainly dependent upon certain personal characteristics, such as prior life experience, and the cultural, social and physical environmental context. There have been several meta-analyses of quantitative studies of postnatal depression (O’Hara and Swain 1996; Beck 1996, 2001). The meta-analysis by Beck (2001) comprising 84 studies identified 13 significant predictors, namely: socioeconomic status, life stress, marital relationship, marital status, self-esteem, history of previous depression, pre-natal anxiety, social support, pre-natal depression, childcare stress, infant temperament, maternity blues, and unplanned/unwanted pregnancy (Beck 2001).

In the scientific field of social epidemiology, there has been a criticism of the paucity of theory and explanation in multilevel studies (Eastwood et al. 2013; Muntaner 1999; Krieger 2001; O’Campo 2003; Carpiano and Daley 2006; Raphael 2006; Kaplan 2004). Shankardass and Dunn (2012) have recently argued for a critical realist approach to identifying “explanatory” mechanisms which often cannot be directly, or empirically, observed. The task in such explanatory research is to identify the mechanisms that produce the observed phenomenon and statistical regularities (Kemp and Holmwood 2003). McEvoy and Richards (2006) observed that “although a combination of quantitative and qualitative approaches is widely advocated, there is considerable scope for confusion due to the complex ontological and epistemological issues that need to be resolved”. The authors argue for adopting the critical realist approach used here to address the problems associated with “paradigm ‘switching’” (McEvoy and Richards 2006).

The aim of the mixed method study reported here was to: (1) describe the phenomenon of postnatal depression in South Western Sydney; and (2) identify mechanisms that would add to our understanding of the psycho-social determinants of maternal depression.

## Methods

### Methodology

Critical realism provided the methodological underpinning for this study. Critical realism assumes ontological and hierarchical stratification of reality (Danermark 2002) making it suitable for the examination of social and psychosocial phenomenon such as socio-economic stratification, social exclusion, isolation and cultural context. We will present here the findings of the qualitative (intensive) study supported by findings from the previously reported quantitative studies (Eastwood et al. 2012a, b).

### Setting

The setting was four local government areas (LGAs) in South Western Sydney, New South Wales, Australia. The area has a diverse multicultural population and is an area of substantial social disadvantage with lower educational attainment and income levels than other parts of NSW. As noted above we have previously reported on clusters of postnatal depression in this region and identified two statistically significant clusters of postnatal depression in northern communities within the study region.

### Quantitative studies

The quantitative study is of 15,389 South Western Sydney mothers who were surveyed, in 2003 and 2004, with the Edinburgh Postnatal Depression Scale (EPDS) (Cox et al. 1987) at the first postpartum home visit. The study utilised the Ingleburn Baby Information System (IBIS) database. The study reports on two outcome variables, namely EPDS >9 and EPDS >12. The IBIS survey contains 45 items which are both clinical (e.g. weight) and parental self-report in nature. As previously reported (Eastwood et al. 2012a, b), forty individual-level variables were selected for exploratory analysis based on prior knowledge, the findings of published research and the findings of the qualitative arm of the study, as reported here.

The exposure variables selected for study were: mother’s country of birth (Australia or other), Aboriginal or Torres Strait Islander culture, age of mother, sex of baby, marital status, household size, blended family, number of children under 5 years of age, accommodation (privately owned or not), employment of mother, employment of father, financial situation (10-point scale), car access, phone access, mother’s rating of her health (five-point scale), mother’s rating of her child’s health (five point scale), breastfeeding (which included both exclusive and partial breastfeeding), smoking, mother’s expectations (“Is being a mother what you expected”—five-point scale), planned pregnancy, previous miscarriage, previous child death, previous stillbirth, previous child disability, previous termination of pregnancy, previous sudden infant death, suburb duration, regret about leaving the suburb (“If for some reason you had to leave this suburb would you be sorry to go?”), support network (“If you had any worries about your child, how many people do you feel you could turn to for help and support, not including health professionals?”), practical support (“Do you receive adequate practical support since the birth of the baby?”), emotional support (“Have you been able to talk to someone about how you are feeling since the birth of the baby?”), mother’s response to her child (“Does the mother respond to the child’s interactions of discomfort?”), mother comforts her child (“Does the mother show the ability to comfort the child?”), mother enjoys contact with the baby (“Does the mother enjoy close physical contact with the child?”), and “Since the birth of your baby how much time did your baby seem: - to have trouble sleeping (five-point scale), to be a demanding baby (five-point scale), to be content (five-point scale), to be a difficult feeder (five-point scale), or to be difficult to comfort (five-point scale)”.

Logistic regression was used for the multivariable analysis (Eastwood et al. 2012a) and the results reported are integrated here. Both the EPDS >9 and EPDS >12 dichotomous outcomes were modelled. Non-linear principal component analysis (PCA) was used to identify latent constructs from the quantitative data for theory generation (Eastwood et al. 2012b). Bi-plots were used to visually present the PCA component loadings as vectors (Fig. 2). The component loadings range between −1 and 1 and indicate the Pearson correlations between the quantified variables and the principal components. The coordinates of the end point of each vector are the loadings of each variable on the two components plotted. Variable vectors that are close together in the plot are closely and positively related. Variables with vectors that make approximately a 180° angle with each other are closely and negatively related. Variables vectors with a 90° angle are not related. The object scores obtained from five identified latent constructs were saved for subsequent logistic regression analysis. All analysis was undertaken in SPSS 19.0 © IBM 2010.

### Qualitative study

The focus groups (FG) were naturally occurring groups consisting of participants of existing “mothers groups”. Previous or current postnatal depression was neither an inclusion nor an exclusion criterion. Three “mothers groups”, with 3–7 members, were purposively selected from: (a) a community with dense housing, low socio-economic circumstances and high numbers of overseas born mothers, (b) a community with predominantly single dwellings, mixed ethnicity and average socio-economic circumstances, and (c) a community with predominantly single dwellings, low socio-economic circumstances and high numbers of overseas born mothers. The groups were facilitated by a research assistant. This avoided coercion and kept researcher distance. Focus group participants could not be identified during analysis. The focus group discussion asked: why they thought some women got depressed during pregnancy or shortly after the delivery of the baby, why there might be more or less depression in some suburbs, what the characteristics of those suburbs might be, and whether there are things at a city, state and national level that might increase or decrease a mother’s depression. Based on emerging themes the third focus group was asked whether certain ethnic groups were likely to have higher rates of depression.

Selection of participants (n = 8) for practitioner interviews was by purposive sampling. Potential participants were identified based on: gender, local government area where they worked, experience with population subgroups, professional or industry background, and emerging concepts. Interview guides for the initial interviews were as for the focus groups. The questions were modified as analysis was undertaken and conceptual theory emerged.

Open coding was the predominant approach taken to initial coding (Charmaz 2006; Saldana 2009). The interview transcripts were coded line-by-line and paragraph-by-paragraph. During the early stages of concept and category generation, each “incident” was coded into as many categories as it might fit, to enable maximum emergence of patterns and relationships (Glaser 1978). A second cycle of coding was undertaken, where the most frequent or significant initial codes were used to develop categories that were more selective and conceptual (Charmaz 2006, p 57). The coding process was considered finalised at the point when there was theoretical saturation. This was the point when no new concepts emerged from reviewing the data from the focus groups and interviews. The data coding, memos and analysis were supported by use of Atlas ti 5.0 software.

A conceptual mapping approach was used to develop causal networks, as described by Miles and Huberman (pp 151–165, 1994). Causal networks display the most important concepts and processes and the relationships among them. The identification of “important” emerging concepts for focused analysis was based on (a) density of coding to that concept or closely related concepts, (b) density of interrelationships and connections among codes, and occasionally (c) abstract abductive and retroductive reasoning. Decisions regarding the nature of the theoretical links between codes were based on (a) information provided by practitioners and mothers, (b) prior knowledge of empirical literature, (c) supporting information arising from concurrent literature reviews and quantitative studies, and (d) abstract abductive and retroductive reasoning.

### Integration

**Fig. 1:**
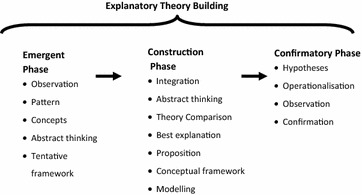
Phases of explanatory theory building

The study reported here was part of a larger concurrent triangulated mixed method design that used an Explanatory Theory Building Method (Fig. 1) (Eastwood et al. 2014).

In the main study integration occurred throughout the study phases with final integration and triangulation occurring in the Construction phase. The study reported here was part of the Emergent Phase where the focus was on observation, pattern recognition, abstract thinking, concept formulation and theory generation. Three methods of theory generation proposed by Haig (2005a, b) are: grounded theory method, exploratory factor analysis and heuristics.

Comparative analysis of quantitative and qualitative findings will be reported here together with limited abstract abductive and retroductive theory generation that utilises the latent constructs identified by our qualitative study and non-linear PCA, as recommended by Haig (2005a, b).

As we have previously reported in this Journal (Eastwood et al. 2014), Haig (2005a) proposed three methods of Theory Generation. They are: Grounded theory method, Exploratory Factor Analysis and Heuristics. In this study we used variants of these methods, namely, emergent theory method and non-linear principal component analysis respectively. Realists distinguish between four modes of inference: deduction, induction, abduction and retroduction. Abduction and retroduction can be defined as follows:

Abduction: To interpret and recontextualise individual phenomena within a conceptual framework or a set of ideas. To be able to understand something in a new way by observing and interpreting this something in a new conceptual framework. Modell (2009, p 213) observes that “abduction does not move directly from empirical observations to theoretical inferences, as is the case in purely inductive research, but relies heavily on theories as mediators for deriving explanations”.

Retroduction: From a description and analysis of concrete phenomena to reconstruct the basic conditions for these phenomena to be what they are. By way of thought operations and counterfactual thinking to argue toward transfactual conditions.

These forms of reasoning are used in the Theory Generation Stage of Realist Explanatory Theory Building to develop tentative conceptual frameworks and to move from the “concrete to the abstract” (Danermark 2002, p 109).

### Ethical matters

Ethics approval was obtained from the University of New South Wales Human Research Ethics Committee. As previously discussed, coercion and disempowerment of focus group participants was avoided by managing the process through the auspices of “third-party” mother’s group organisers. Participation was voluntary, the purpose was made clear, questions were invited, benefits explained and signatures of both the researcher and participant obtained after there was agreement to participate. The “mothers group” participants were deliberately not drawn from currently stigmatised communities or neighbourhoods. The focus group members knew each other and signed a confidentiality form declaring that all details of the focus group participants and discussion would be kept confidential. The privacy and anonymity of the participants was maintained and the data will be destroyed after 7 years. The information regarding the study also included information that had previously been distributed as part of national and local perinatal depression initiatives. There was a remote possibility that participants could have been distressed, by discussing a topic such as perinatal depression. For this reason information was provided to all participants about postnatal depression with contact details for relevant health care providers. Additionally the facilitator was an experienced general practitioner, who was alert to the possibility of participants becoming distressed during the focus group.

## Results

The qualitative (intensive study) results are reported thematically below with previously reported quantitative results integrated as appropriate. We identified ten theoretical concepts that might explain the psycho-social mechanisms contributing to maternal depressive symptoms in our community. They were: attachment and nurturing, infant temperament, unplanned pregnancy and sole parents, support for mothers, access to services, stress, financial hardship, isolation and marginalisation, mothers “loss of control” and “power, and expectations and dreams.

### Attachment and nurturing

Attachment and nurturing were not initially selected as prior concepts but emerged very early in both the practitioner interviews and literature review. Most of the practitioners and mothers’ groups stressed the link between postnatal depression and poor attachment and nurturing of the infant. One practitioner described depression as a veil that prevents a mother from relating to the infant, understanding the baby’s cues, and connecting to the baby as an individual and as a person. The lack of eye contact was seen as important by another contributor together with “lack of appropriate affect, less interaction and less intellectual stimulation”.

A further consequence of poor maternal attachment was that “you will have a restless baby and insecure attachment which would thus affect the infants’ temperament” (Practitioner JH). The negative impact of altered infant temperament on postnatal depression was commented on by several mothers as setting up a complex situation where infant temperament may be a cause of postnatal depression and postnatal depression may itself contribute to altered infant temperament.

Family “dysfunction” and “chaos” was also described as interfering with attachment. In this situation the “baby gets lost sight of in the whole thing” (Practitioner LC). The chaos in the family and other things going on affect how people relate to the infant. Practitioners felt that in this situation the physical care of the infant may be satisfactory but the need for psychological interaction “can be overlooked”.

The importance of parent-infant attachment is well established in contemporary developmental psychology as a “development pathway of major significance throughout the child’s life course” (Swain et al. 2007). Postnatal depression has consistently been shown to impact negatively on maternal-infant attachment (Martins and Gaffan 2000). Non-linear principal components analysis of the quantitative arm of the study (Eastwood et al. 2012b) identified a latent variable which we called *maternal responsiveness* (Cronbach’s Alpha = 0.79). As this latent construct was determined to be an outcome of maternal depressive symptoms it was not modelled in the logistic regressions.

### Infant temperament

The role that infant temperament might play in postnatal depression emerged early from both practitioner and mothers groups interviews. Mothers spoke of the difficulties faced when babies were not sleeping, crying or behaving in ways that they did not expect. One mother spoke of being so depressed and upset at everything because “I can’t get help with whatever he is doing, or he is crying” (FG 1), extend quote). Another spoke of the reality of lack of sleep, “not being able to control was he is going to do”, not being able to make the baby stop crying and consequently “wanting to cry” (FG 3).

Infant temperament has been defined as the infant’s behavioural style and is how they behave in relationship to the environment and caregiving that they receive (Thomas and Chess 1977) cited by (McGrath et al. 2008). As discussed above a consequence of poor maternal attachment was that “you will have a restless baby and insecure attachment” (Practitioner JM). This then sets up a complex situation where infant temperament may be a cause of postnatal depression (and poor attachment) which may further contribute to altered infant temperament.

**Fig. 2:**
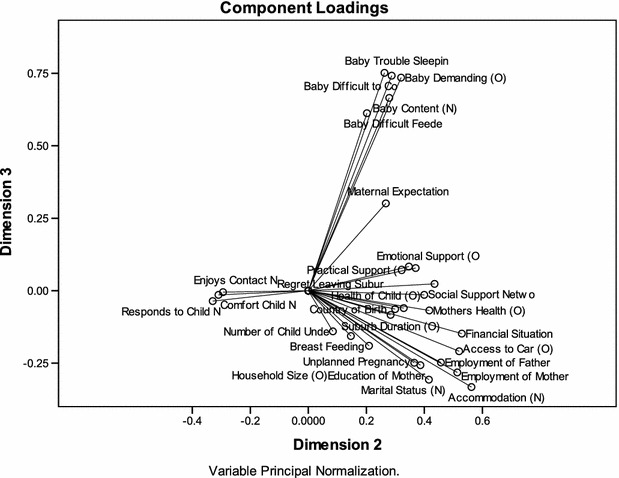
Bi Plot of dimensions 2 and 3

Five variables measuring aspects of infant temperament were included in the quantitative analysis. The variables *baby difficult sleeping* and *baby demanding* independently predicted maternal depressive symptoms (EPDS >9 and >12) in our multivariate logistic regression (Table 1) (Eastwood et al. 2012a). In the non-linear PCA (Table 2) the five variables loaded on dimension 3 (Fig. 2) which we called *infant behaviour* (Cronbach’s Alpha = 0.72). The dimension, or latent variable, *infant behaviour* predicted postnatal depressive symptoms (EPDS >12) in the multivariable logistic regression (OR 1.14; 95 % CI 1.10–1.20) (Eastwood et al. 2012b).

**Table 1:** Final parsimonious model EDS >9

	B	SE	Wald	*df*	Sig.	OR	95 % CI for OR	
							Lower	Upper
Maternal expectation (O)	0.642	0.035	338.182	1	0.000	1.901	1.775	2.036
Baby difficult sleep (O)	0.138	0.042	11.032	1	0.001	1.148	1.058	1.246
Baby demanding (O)	0.092	0.038	5.975	1	0.015	1.096	1.018	1.180
Baby not content (O)	0.171	0.048	12.534	1	0.000	1.186	1.079	1.304
Practical support (D)	0.205	0.075	7.481	1	0.006	1.228	1.060	1.422
Emotional support (D)	0.446	0.083	29.109	1	0.000	1.562	1.328	1.837
Social support (O)	0.149	0.028	28.040	1	0.000	1.161	1.099	1.227
Financial situation (O)	0.086	0.017	25.094	1	0.000	1.090	1.054	1.127
Country of birth (D)	0.225	0.058	15.070	1	0.000	1.252	1.118	1.402
Mothers health (O)	0.365	0.037	98.047	1	0.000	1.440	1.340	1.548
Constant	−6.216	0.178	1213.109	1	0.000	0.002

**Table 2:** Component loadings for five dimensions

Variable	Dimension				
	1	2	3	4	5
Responds to child	*1.148*	−0.336			
Comfort child	*1.129*	−0.316			
Enjoys contact	*1.103*	−0.300			
Accommodation		*0.560*	−0.334	−0.343	
Financial situation		*0.528*			
Access to car		*0.518*			
Employment of mother		*0.512*			
Employment of father		*0.455*			
Social support network		*0.435*		*0.424*	
Mothers health		*0.421*		*0.421*	
Marital status		*0.413*	−0.308	*−0.562*	
Country of birth				*0.527*	
Health of child		0.400		*0.492*	
Education of mother		0.386			
Emotional support		0.370			
Unplanned pregnancy		0.363		−0.344	
Practical support		0.345			
Regret leaving suburb		0.328			
Baby trouble sleeping			*0.751*		
Baby difficult to comfort			*0.741*		
Baby demanding		0.320	*0.735*		
Baby difficult feeder			*0.612*		
Baby content			*0.664*		
Number of children under 5					*0.746*
Household size					*0.634*
Suburb duration					
Maternal expectations			0.301		
Breast feeding					
Total (Eigenvalue)	4.16	3.67	3.21	1.91	1.38
Cronbach’s alpha	0.79	0.75	0.72	0.48	0.28
Percentage of variance	14	13	11.5	6.8	4.9

Component loadings greater than 0.40 are in italic and loadings less that 0.30 are suppressed

### Unplanned pregnancy and sole parents

The phenomenon of unplanned pregnancy in relation to maternal depression was thought to be significant particularly if the pregnancy was unwanted. Also important was the implication that the mothers might be unsupported. Cultural issues were considered to be important with some parents not giving support to their pregnant daughters if they were not married. Practitioners felt that the degree of support given may depend on the nature of the communities that mothers lived in. In some cultures there was a lot of support and the family belief would be “oh my god, you are pregnant, this is god’s gift to the family” (Practitioner SH). It was also considered possible that “unsupportive cultural beliefs and attitudes may also exist within some mainstream cultures”. (Practitioner VN).

Mothers in the focus groups also spoke of the impact of an unplanned pregnancy on their relationship with their partner and on their mental preparedness. In this situation the partner may not be prepared and although they are with them they are not supportive. In some cases the relationship changes. Mothers were clear that “the mother is always seen as the one that bears it all”. (FG 2).

Links to social isolation were also made. Mothers felt it would be hard for sole parents who didn’t have an education to find a job or get to TAFE (technical and further education college). They felt that these mothers would not have the “social skills to make friends with nice people, or even have someone to look after your baby while they go to TAFE and try and educate yourself” (FG 2). Unplanned pregnancy, low income and poor education were seen to be associated with staying home and not being able to network with friends. One mother noted that:

“If you have no one there, you are stuck. You have to stay at home with them. If you don’t have the money to go out for lunch with friends. Like that is a big thing for me, I have to be able to go out and see my friends have lunch with them or whatever. I have to get out of the house at least once every day. If you don’t have the money to go and do that then I guess you will feel a bit snowed under.” (FG 1)

Unplanned pregnancy and sole parenthood were thus seen by both practitioners and mothers as a cause of both antenatal and postnatal depression.

In the quantitative arm of the study, sole parenthood was independently predictive of EPDS >12 (OR 1.56; 95 % CI 1.25–1.95) (Eastwood et al. 2012a). This finding is consistent with extant literature (O’Hara and Swain 1996; Beck 2001). In the non-linear PCA sole parenthood also loaded on latent variables that we called *social exclusion* and *migrant isolation* (Table 2) (Eastwood et al. 2012b) The negative loading on *migrant isolation* suggests that migrant sole parents are protected. The mechanism may be related to cultural beliefs and attitudes of migrant communities toward sole parents.

### Support for mothers

The care and support of mothers was a strong theme emerging from both practitioners and mothers groups. The construct *Support for Mothers* had a number of sub-categories including family support, partner support, emotional support, and support from services through play groups, visiting nurses or midwives and phone calls. We can only highlight selected aspects of the rich qualitative data here.

Support networks were mentioned by both practitioners and mothers and usually included family members and friends. As discussed above the care and support of mothers differs among families and may be related to whether she is partnered. One practitioner noted that “a lot of young mothers who get pregnant don’t have that support from their partners or their parents. So it depends what level of support there is.” (Practitioner TC)

The mother’s spoke of the importance of their partner being there for them and also wondered what it must be like if the mother was a single parent. One mother expressed this as:

“but even then I find it, looking at me, I go then, thank God Todd’s with me, so he can give me the hug, during the night when I am so depressed you are really upset about everything because I can’t get help with whatever he is doing or he is crying or whatever. Then I think a single parent, I go how do you do it?” (FG 2)

The mothers placed strong importance on the importance of the groups for establishing and maintaining support networks. One mother noted that support networks may include family, friends and neighbours—“I think just having support networks whether it is something like this or whether it is your family, friends, your next door neighbour, again even if you have all those things it is no guarantee that you won’t suffer from PND” (FG 3)

The parent groups provide a useful support network. One mother stressed their importance and linked this to filling a gap when there is lack of support available from family—“…more groups. This is fantastic. I didn’t have a group like this when I had my first, and didn’t have support. You are crying for… family and that sort of thing. Having these networks has helped me a lot more.” (FG 1)

There is strong literature support for the protective role of partner support and social networks (Beck 2002a). Our quantitative studies also found lack of emotional support and social support to be independently predictive of EPDS >9 (OR 1.6; 95 % CI 1.3–1.8) and (OR 1.2; 95 % CI 1.1–1.2) respectively (Table 1) (Eastwood et al. 2012a).

### Access to services

The construct *Access to services* included access to information, car and phones. Living in a community with few amenities or services was considered a group level phenomenon and has been previously reported (Saldana 2009).

Some mothers may lack information and it was felt by mothers that it was governments’ role to give mothers access to that information. A mother in a mothers group felt that “people aren’t aware of what is available. So may be government needs to let any mother know what type of treatment is available. I don’t know of any type of treatment yet. We are not aware of it. And whether we can access it or not”. (FG 3)

Another mother felt that access to books and the internet was important. The role of mothers groups and friends for sharing information was raised by one mother—“talking to other people you know who are going through the same thing … but when you are in a group and actually get talking” (FG 3). The importance of information was supported by a practitioner who when discussing the importance of linking to services noted that “not all of them have the knowledge to be linked” (Practitioner JH).

One of the mothers linked transport problems with isolation “I think transport problem is significant, it is hard to get out and about,—I suppose there is isolation”. (FG 1) This view was supported by four practitioners who spoke of the importance of having cars for “getting out to other people”. Public transport was seen as a possible barrier—“If it is just an effort if you have baby and a toddler and have to pack up a pram and wait an hour for a bus to go somewhere. Then you can only do limited shopping, you have to carry it home” (FG 1). Another practitioner felt that there was an expectation that people have cars. She had similar concerns regarding the effort to pack up “babies and kids” and take them out.

Access to a phone was considered important to mothers and that phone may be at a neighbour’s phone. One mother stated—“or you speak to someone you know, who has just rung them. That is what I have done. I haven’t rung myself yet but I keep asking a woman who does” (FG 3). Another mother recalled “… a lovely lady at three in the morning, after my son had this massive projectile vomit at about 2 weeks. Something out of a horror movie, I thought this is not right. Yes, just to be able to talk to her at that time knowing that somebody was there. … Even when I was crying to them on the phone they were so supportive and sympathetic.” (FG 2)

In our quantitative studies access to cars and phones was correlated with depressive symptoms but was not significantly associated in the final adjusted models. In the non-linear PCA, access to a car loaded on the latent construct *social exclusion* which was independently associated with EPDS >12 (OR 1.8; 95 % 1.7–1.9) (Table 3) (Eastwood et al. 2012b). We were not able to study here the impact of child and family nursing services on postnatal depression but other studies have found service interventions to be protective (Dennis 2005; Morrell et al. 2009).

**Table 3:** Univariate and multivariable logistic regression on EPDS >12

	B	SE	Wald	*df*	Sig.	Exp (B)	95 % CI for EXP (B)	
							Lower	Upper
Univariate logistic regressions								
F2-Social exclusion	0.732	0.032	531.541	1	0.000	2.079	1.954	2.212
F3-Infant behaviour	0.326	0.029	125.575	1	0.000	1.385	1.308	1.466
F4-Migrant isolation	0.301	0.034	79.949	1	0.000	1.351	1.265	1.443
F5-Large household	−0.122	0.034	12.550	1	0.000	0.885	0.828	0.947
Maternal expectations	1.019	0.042	576.893	1	0.000	2.771	2.550	3.012
Multivariate logistic regression model								
F2-Social exclusion	0.586	0.035	285.278	1	0.000	1.797	1.679	1.923
F3-Infant behaviour	0.127	0.031	17.198	1	0.000	1.135	1.069	1.205
F4-Migrant isolation	0.206	0.033	39.963	1	0.000	1.229	1.153	1.310
F5-Large household	−0.030	0.033	0.831	1	0.362	0.970	0.909	1.036
Maternal expectations	0.718	0.047	228.817	1	0.000	2.051	1.869	2.251
Constant	−5.085	0.166	939.442	1	0.000	0.006		

### Stress

Previous studies of postnatal depression have consistently found stressful life events to be risk factors and few studies have found no association. It was not surprising therefore, that the phenomenon of stress was mentioned by most practitioners and was strongly implicit in responses from the mothers groups.

The term stress was only mentioned once by a mother when speaking of postnatal depression when she said that “you have more stresses in your life, financial, relationships, loss of social income”(FG 1). We considered that stress was often being talked about when mothers were talking of “losing control”, “crying over the phone”, “something out of a horror movie”, and “I am going to be graded”.

Some of the predictors identified by practitioners and mothers included: lack of support, poverty, isolation, living in “depressed communities”, conflicting advice, domestic violence, drug problems, fractured relationships, arranged marriage, pregnancy, traumatic experience, loss of family members, working and travel to work. As discussed elsewhere, support was felt to reduce stress.

Adversity from whatever source causes stress and was linked to depression. Both practitioners and mothers spoke of a wide range of causes of adversity including cost of living, crowding, violence, crime, and drug problems. As discussed above there was a link between stress and the themes of marginalisation, isolation and loss of control.

We did not have measures of stress in this study. The phenomenon of perinatal depression has, however, repeatedly been found to be associated with stressful life events (O’Hara and Swain 1996; Beck 1996, 2001; NHMRC 2000; Wilson et al. 1996). That depression is caused by psychological stress is increasingly certain (Kinderman 2005; Stone et al. 2008) but it is less clear whether stress is a necessary condition that must be present for there to be depression.

### Financial hardship

Both practitioners and mothers spoke of financial hardship as a cause of postnatal depression. Mothers were very clear that postnatal depression could be the result of “being broke”, “economic factors”, “their income”. Mothers spoke of not being able to provide as much for their baby, “things like nappies and formula and that type of thing” (FG 1). Mothers spoke of the struggle to cover basic living needs after the baby was born. Their partners would look to “pick up extra overtime” to cover mothers being off work. The financial stress of losing an income meant that many would struggle to cover the mortgage, rent, food and “basic living”.

The struggle to cope was clearly a cause of stress for the mothers. When asked if there was anything that government could do to assist, one mother suggested “give us a house cleaner once a week – someone to clean my shower” (FG 2). They did not want to be “bludging off government” but strongly felt that family assistance should be for everyone. The stress for many mothers was related to living beyond their means at the time the baby was born.

Sole parents were considered to particularly vulnerable often having no transport, less money, living in disadvantaged suburbs and not being able to “buy happiness”. Financial resources were considered to “play a huge part in anyone’s system” (Practitioner VN). If mothers don’t have money, they don’t where the next meal is coming from, are struggling to pay the rent, fill the car with petrol and “juggling to get to places” (Practitioner GA).

These findings are consistent with extant literature and we found that family financial situation is weakly predictive of EPDS >9 (OR 1.1; 95 % 1.05–1.13) and EPDS >12 (OR 1.14; 95 % (1.08–1.19) (Eastwood et al. 2012a). Financial situation also loaded on the latent variable *social exclusion* which was predictive of EPDS >12 (OR 1.8; 95 % 1.68–1.92) (Table 3) (Eastwood et al. 2012b).

### Isolation and marginalisation

The theme of *Isolation* and having “no support” were closely related. Related was the concept of *marginalisation* which one practitioner felt that “is [the] big one.” By marginalisation they described the mother as not being “accepted as part of main stream with a link between marginalisation, stress and depressive symptoms” (Practitioner VN).

Although *marginalisation* was not used by mothers, there was a consistent use of “isolation” to describe a similar phenomenon. Mothers spoke of isolation when a mother was not of the same language group—“if they don’t know your language they [are] just isolated” (FG 3).

Marginalisation was also considered to occur by being isolated through the impact of poverty, sole parenthood and lack of transport. One practitioner when speaking of the impact of poverty described the impact as something other than survival. “Not survival it’s something else. It’s just marginalised being so disadvantaged” (Practitioner LC).

Isolation was spoken of by several practitioner and mothers as relating to family relationships and in particular lack of support. “Again I am going to hype back to relationships. Is she isolated? Is she vulnerable? Is this a single pregnancy?” (Practitioner VN)

Community safety emerged as sub-category for isolation. Practitioners spoke of the isolation that can occur as a result of not feeling safe in a community. Mothers were also concerned about community safety and linked it to isolation—“If you want to go the park for example and there are some violent people then you choose not to go to the park. Then you are isolated.”(FG 1)

The isolation experienced by mothers may be experienced by those not living in poverty or marginalised by society. One practitioner spoke of areas where they do not feel welcome or in a new area away from their parents or usual “social base”. Some of these mothers may be career women who have had high expectations who suddenly with the new baby have “things crashing on top of them and not going the way they want. They don’t have social support, they don’t know the people in the neighbourhood and things can isolate them even further”. (Practitioner JH)

Isolation would seem to be the result of a number of processes including marginalisation from the group for social, language or cultural reasons, social exclusion as a result of poverty and sole parenthood, and isolation as a result of lack of transport. Taken together these concepts relate to being “alone” and “unsupported”. As noted above there is a strong extant literature related to the role of partner support and social networks and our quantitative studies found a lack of emotional and social support to be independently predictive of EPDS >12. The latent concepts *social exclusion* and *migrant isolation* were also predictive of EPDS >12 (OR 1.8; 95 % CI 1.68–1.92) and (OR 1.2; 95 % CI 1.15–1.31) respectively (Table 3) (Eastwood et al. 2012b).

### Mothers “loss of control” and “power”

A number of practitioners and mothers themselves spoke of “loss of control”. This “loss of control” was felt to contribute to the development of depressive symptoms. Three main sub-categories were identified related to personal relationships, birth of a new baby and conflicting advice. There was also an association between “loss of control of your life” and “expectations lost”. The construct “Expectations lost” emerged as an important theme in subsequent analysis.

Practitioner’s spoke of “loss of control” related to whether the mother was supported or “unsupported” in her personal relationship. They expressed it as “Not purely but mostly feeling safe healthy supported and connected”. For mothers the birth of new baby brought with it a loss of control often resulting in changes in their relationship with their partner. The loss of control was expressed as a complete change in their sense of personal self-control—“once I had the baby I didn’t have that control and that affected me the most”. (Practitioner SH)

Mothers spoke of a sense of letting themselves go and not dealing with their own personal needs. Consequently “things started to build up”. One mother spoke of “complete loss of control of your life”. She reflected that it is not possible to prepare emotionally for the loss of control when she had previously in control of her life and able to plan ahead. With the birth of the baby “now you can forget it”. (FG 2)

Advice given to mothers by “western” trained workers sometimes conflicted with advice that mothers received from their own communities. One practitioner identified this as cause of stress for mothers. She spoke of the “individual strengths” needed by mothers to stand up for what they wanted for themselves and their infants. Speaking of her personal experience with mothers she identified increased stress at home when mothers didn’t have the “power to say that this is what I want for my child”. In these situation she felt that mothers didn’t know who to listen to and they didn’t have the “individual strengths to stand for what they want or they don’t know how to form their own judgement [about] what is best for their child”. (Practitioner JH)

Also evident in the discursive elements was the lack, or absence, of power that mothers have. One practitioner commented that:

“there are some women who have social expectations which are well above what the society can let them have.” (Practitioner JM)

When discussing mothers in ethnic communities one practitioner spoke of the lack of power that mothers may have in relation to themselves and their baby.

“and lots them they don’t have the individual strengths to stand for what they want or they don’t know how to form their own judgement - what is best for their child or they even have the power. But lots of them … they don’t have the power to say that is what I want for my child”. (Practitioner SH)

The “loss of power” and “loss of control” is also implicit in comments about career women who were high achievers with high expectations when they were working

“suddenly with a baby and things are crashing in on top of them and it is not going the way they want. They don’t have the social support [and] they don’t know the people in the neighbourhood, … I guess they lock themselves into a mental state where they need some help” (Practitioner JM)

We were not able to quantitatively measure this construct but note that Beck identified loss of control as the basic social psychological problem that postnatal depressed mothers have to contend with (Beck 1993). In a subsequent meta-synthesis Beck (2002b) confirmed that this loss permeated deep into the lives of depressed mothers.

### Expectations and dreams

We noted above that shattered maternal expectations may be important. Absent was discussion of the dreams that mothers might have. Yet implicit in comments by mothers were their dreams for the perfect baby, home and future.

When discussing why mothers may get depressed one mother talked of the rude shock when motherhood was not as portrayed in the “Huggies adverts”. The reality was that her baby would not stop crying. She spoke of the frustration of not knowing what to do after checking the “three things they told me to check”. She had the expectation that when she got home she would know what to do and “it will be all good”. But the reality was that “it doesn’t work like that”. (FG 2)

Both mothers and practitioners felt that society and mothers expected to take home the perfect baby and that everything was “going to be fine”. The reality is often different to that portrayed in media such as the “Huggies adverts”. The shattering of dreams and expectations was described in a range of different ways.

For mothers with few resources their hopes for the future may be dashed by the impact of the new baby on both financial and emotional resources. This may be especially true for a young woman who had plans for her life. The reality is different as expressed by one practitioner.

“[Parenting] takes a great deal of emotional energy and to do it well, to be available for children and provide all their needs. Parents that are distracted by adverse environmental factors such as finance or domestic violence don’t have that energy” (Practitioner LC)

Mothers “Loss of control” was discussed previously. Mothers implied that they had been “in control” of the lives (and also their husbands) and “expected” to be in control after the baby was born. But this was not always the case and several mothers when speaking of the “loss of control” implied that this was not their expectation of how motherhood was going to be. With the birth of the baby planning ahead was no longer possible.

Society has expectations of what a “good mother” will be like as discussed in relation to the “Huggies Advert”. One practitioner felt that some mothers “don’t think they are doing a good job”. Mothers agreed with this expectation. Some spoke of expectations raised by helping services such as midwives. One mother talked of the stress associated with the nurse visit.

“Now I have to get myself ready, make sure I don’t look like I have …., so they don’t think I am a bad mother” (FG 1)

Discussion about the home visit also focused on expectations related to “you are not bonding with your child and you are expected to”, and “I am going to be graded, … please don’t put me in the high risk”. (FG 1)

One practitioner spoke of “career women losing it”. Several practitioners agreed with the mothers who had spoken of the shattering of expectations of what motherhood would be like. One practitioner put it like this

“Career women that have been working, who have had high expectations when they have been working high achievers in their career, suddenly with a baby thing are crashing in on top of them and it is not going the way they want.” (Practitioner JM)

In her meta-synthesis of 18 qualitative studies Beck (2002b) identified “incongruity between expectations and the reality of motherhood” as one of four overarching themes.

In the quantitative logistic regression study maternal expectations of motherhood was a strong independent predictor of maternal depressive symptoms—EPDS >9 (OR 1.9; 95 % CI 1.8–2.0), EPDS >12 (OR 2.1; 95 % CI 1.9–2.3) (Eastwood et al. 2012a). In the non-linear PCA the variable *maternal expectation* had a long vector in the bi-plots indicating that it accounted for a large amount of variance (Fig. 2). It did not, however, load onto one of the five dimensions. Consequently we elected to include *maternal expectation* in the related regression analysis as an empirical variable. *Maternal expectation* was strongly predictive of EPDS >12 (OR 2.0; 95 % CI 1.9–2.2) (Eastwood et al. 2012b).

## Discussion

As we noted above psychological stress is increasingly being recognised as certain cause of depression (Kinderman 2005; Stone et al. 2008). The task in explanatory research is to find the, often unobserved, mechanisms that produce the actual phenomenon. Our empirical quantitative and qualitative data has identified a large number of possible factors including: financial stress, being born outside Australia, infant behaviour and lack of emotional support. But emerging from our data are the two central mechanisms:

### Lost expectations

#### Being alone

The empirical variable *Maternal Expectation* consistently has an adjusted odds ratio of 2.0. Visual examination the Bi Plot for Dimensions 2 and 3 of the PCA reveals *Maternal Expectation* is midway between constructs of *Infant behaviour* and *Social Exclusion* (and *Migrant Isolation)* (Fig. 2). This juxtaposition supports the proposition that expectations are lost in relation to (a) infant behaviour, and (b) social exclusion (or alternatively social inclusion). Also evident is the strong influence of the latent construct *Social Exclusion* with an adjusted odds ratio of 1.8 and the isolation of migrant mothers. These findings are consistent with the stories of motherhood not meeting expectations.

Also buried in the voices of the mothers were stories of “being alone” with the crying infant, with an absent partner, mother or other support friend. Sometimes mothers spoke of not being able to get out to see their friends. The descriptions were powerful and suggested that this situation is experienced by many mothers.

We agree, therefore, with Beck (1992, 1993, 2002b) that depressed mothers experience an “incongruity between expectations and the reality of motherhood”, a sense of pervasive loss, and loneliness.

One hallmark of critical realist analysis is the ontological assumption that reality consists of hierarchically ordered levels where a lower level creates the conditions for a higher level. The higher level is not, however, determined by the lower level and has its own “generative mechanisms”. The existence of these level specific generative mechanisms is what constitutes or defines a level. The implication of this stratification is that it is not possible to reduce the causes of what occurs on one level to those on another level (whether lower or higher) (Danermark and Gellerstedt 2004).

Possible hierarchical structures, mechanisms, contexts and outcomes are shown below (Table 4) based on the findings of this study, and our previously reported ecological analysis (Eastwood et al. 2014).

**Table 4:** Analytical levels of depression and context

Levels	Example of structures	Example of mechanisms	Example of contexts	Example of negative outcomes
Global economic	Multinational companies	Exploitation, profit	Advertising	Huggies advert of ideal motherhood
Cultural	Ethnic community	Segregation	Migration	Excluded by bonding networks
Social	Neighbourhood social capital	Relationships	Social networks	Isolation
Social	Family	Emotional support	Absent partner	Isolation
Psychological	Self	Relation to self	Isolation	Feeling overwhelmed and alone
Psychological	Self	Relation to self	Cannot sooth crying infant	Expectations of motherhood are lost
Psychological	Mind	Stress	Overwhelmed and alone	Depression, reduced motivation, anxiety
Biological	Body	Neurobiological	Reduced motivation	Hypoactivity of motivation areas

The above approach is useful as a theoretical analytical framework but “in reality levels are entwined and [the] mechanisms could be supporting each other or counteracting each other, and the outcome in a specific context is the result of a very complex interplay between levels and mechanisms” (Danermark and Gellerstedt 2004).

### Methodological matters

We have previously reported the strengths and limitations of the quantitative (extensive) (Eastwood et al. 2012a, b, 2013) and qualitative (intensive) methods (Eastwood et al. 2014) used in this study.

As we have previously observed in this Journal, the size (15,389) of this cross-sectional study of the EPDS administered to postnatal women is unique. There have been few previous reports of postnatal depression studies on population samples of this order. We have also previously reported on the limitations of the cross-sectional design for causal inference and the impact on generalizability of sample selection bias from refusal and non-response in the study population. Importantly not all households with births in the study period were surveyed (Eastwood et al. 2014).

The strength of the qualitative (intensive) methods used in this study is their ability to provide explanatory power to the analysis (Sayer 2000) and to identify possible causal mechanisms. The quality of the qualitative study was assessed using criteria that differ from those used for extensive quantitative studies (Kitto et al. 2008; Greenhalgh and Taylor 1997; Miyata and Kai 2009; Cohen and Crabtree 2008). The procedural rigour was made explicit through clear articulation of the ontological and epistemology position informing the study; the sampling techniques used was purposeful and sought to include subjects from different communities, and ethnic backgrounds; and Interpretive rigor was enhanced by integrative and synergistic use of both quantitative and qualitative data. The number of mothers groups interviewed was limited in this study to three which may limit generalizability. Despite these limitations the qualitative data was rich and contributed significantly to both theory generation and later theory construction. It was notable that the findings from this qualitative study are similar to those found by Beck in both her original phenomenological (Beck 1992) and grounded theory (Beck 1993) studies and in her later meta-synthesis (Beck 2002b).

Missing from this study has been an intensive study of the experiences of depressed mothers. While the findings are consistent with previous qualitative studies, further confirmation and understanding of the phenomenon, can be gained from case studies with mothers who have, or are experiencing postnatal depression. Such case studies could be used to examine the propositions arising from this study and should include migrant women, young unsupported mothers, mothers in depressed neighbourhoods and those living in suburbs with strong aggregated social networks.

The findings of this study have implications for health service and public health interventions. Such interventions should focus on addressing the isolation and marginalisation experienced by the above groups of mothers. Those interventions might include intensive social support and integrated care components. The evaluation of those initiatives would benefit from using realist programme theory and evaluation methods as described by Pawson and Tilley (1997).

## Conclusion

Emerging from this mixed method study is the centrality of *being alone* and *expectations lost* as possible triggers of stress and depression for mothers. These findings might also apply to others who have their dreams shattered during life’s transitions. In these situations social and cultural context can either nurture and support or marginalise and isolate.

The challenge for policy and practice is to support mothers and their partners during the transition to parenthood within a challenging social and material context.

## Abbreviations

EPDS: Edinburgh Postnatal Depression Scale
LGA: local government areas
EDA: exploratory data analysis
IBIS: Ingleburn Baby Information System
PCA: principal component analysis
OR: odds ratio
CI: confidence interval
ROC: receiver operating characteristics
DAG: directed acyclic graphs
TAFE: technical and further education
PND: postnatal depression
